# Associations between paid and unpaid work among Norwegian seniors: competition, complementarity or continuity?

**DOI:** 10.1007/s10433-021-00615-9

**Published:** 2021-03-22

**Authors:** Hanna Vangen, Tale Hellevik, Katharina Herlofson

**Affiliations:** NOVA, OsloMet – Oslo Metropolitan University, Stensberggata 26, 0170 Oslo, Norway

**Keywords:** Active ageing, Informal help and care, Formal volunteering, Older workers

## Abstract

A key issue in policy debates on active ageing is how to increase older people’s participation in both paid and unpaid work. This combined goal raises the question of whether the different activities compete for seniors’ time and energy or whether it is possible to achieve both, since such activities may instead complement one another. To address this issue, we examine associations between paid work, informal help provision and formal volunteering among 62- to 75-year-olds by using longitudinal data from the Norwegian Life Course, Ageing and Generation Study (2007, 2017). Our analyses show that both work exit and part-time work are associated with a higher probability of doing unpaid work in senior years compared with full-time work. However, previous engagement in unpaid activities matters considerably, regardless of paid work status. Individuals involved in informal help or formal voluntary work in 2007 were far more likely to do unpaid work 10 years later than those who were not involved. Since seniors who are already engaged in unpaid activities before leaving the labour market are likely to continue to provide informal help and volunteer, we argue that initiatives to stimulate combinations of paid and unpaid work in late careers may be advantageous.

## Introduction

Over the past decade, several initiatives have been launched to stimulate the participation of older people in society. Examples include the European Year of Active Ageing and Solidarity between Generations in 2012 (European Commission 2010), ‘Live Longer, Work Longer’ (OECD 2006) and ‘Towards an Age-friendly World’ (WHO 2015). In Norway, the most important effort is the government’s recent reform entitled ‘A full life—all your life’ (Norwegian Ministry of Health and Care Services 2019), which aims to create a more age-friendly society in which older people participate in the community and live meaningful lives. The initiatives build on the idea of ‘active ageing’, a concept that can be traced back to earlier contributions in social gerontology, such as ‘activity theory’ (Havighurst 1961) and ‘continuity theory’ (Atchley 1989), as well as theories of ‘productive ageing’ (Butler and Gleason 1985) and ‘successful ageing’ (Rowe and Kahn 1987). All these approaches to ageing stress the importance of continued activity and involvement for older people’s well-being and health.

Towards the end of the 1990s, due to demographic changes, with increasing population ageing, the idea of ‘active ageing’ made its move from science to policy, and particularly in the European debate, to a stronger emphasis on the contribution of active older people to the sustainability of the welfare state (Walker 2016). Although policy discussions have tended to concentrate on paid employment, active ageing is far from solely about extending working life (Foster and Walker 2015). In fact, the joint European initiative, the Active Ageing Index, clearly recognises the central role of unpaid activities like formal volunteering and informal help and care provision (European Centre Vienna 2013). An essential question then becomes whether the different activities compete, i.e. if participation in one activity takes place at the expense of involvement in another, or whether they complement one another. Since time is a finite resource, one might assume the former (competition or trade-off) to be the case. On the other hand, individuals who are engaged in one type of activity may be more likely to participate in others, implying that there exists a ‘more-is-more’ (or complementarity) phenomenon. So far, findings are inconclusive as to which mechanism is more prominent. To better understand how the various activities are related, we will employ longitudinal data from the Norwegian Life Course, Ageing and Generation Study (Veenstra et al. 2021). These data also enable us to address the importance of continuity by examining whether current participation in unpaid work depends on earlier involvement in the same activity or whether engagement in paid work is likely to be replaced by engagement in unpaid activities later in life.

## Background

### Theoretical context

The literature offers various theories on how productive activities (paid and unpaid) may be linked in later life, some pointing towards a competing/trade-off relationship (engagement in one activity *decreases* the likelihood of being engaged in others), others implying a complementary/more-is-more relationship (engagement in one activity *increases* the likelihood of being engaged in others). A negative relationship would be the outcome of ‘role overload’ or ‘role strain’ (Goode 1960). If different activities compete for individuals’ time, energy and/or commitment, starting a new activity is likely to result in a need to reduce participation elsewhere, whereas quitting an activity leaves more room for other types of engagement. A second proposed mechanism leading to a negative association is ‘role substitution’, a tendency for individuals to feel role loss when they leave an activity (commonly paid work for older individuals) and then to compensate for this loss by taking up other activities (Chambré 1984; Herzog et al. 1989). A positive association, on the other hand, is a probable outcome if involvement in one activity increases the opportunity or motivation to participate on other arenas, referred to as ‘role extension’ (or the more-is-more principle) (Hank and Stuck 2008; van der Horst et al. 2016). Another argument used in support of a complementary relationship is that engagement in a productive activity is vitalising and creates energy. Consequently, instead of multiple activities being in conflict, it is more the case that activity breeds activity (Marks 1977). This line of argument also implies that exit from paid work is accompanied by decreased engagement in other activities (Mutchler et al. 2003).

Being active in older age may depend on earlier activity patterns. Of relevance here is the concept of continuity, which can have different aspects: continuity within the same activity, meaning that people engaged in an activity are likely to continue being engaged (Mutchler et al. 2003), and continuity in the sense of leading an active life in general. The latter implies that if an activity is interrupted by, for example, retirement, the lost activity may be substituted by another (e.g. volunteering). This aspect is in line with the role substitution mechanism, as well as with Atchleys’ (1989) continuity theory stating that people adapt to changes associated with ageing by taking part in activities that confirm a sense of continuous self-identity across different social domains.

### Earlier research

The association between paid and unpaid work has been analysed in several previous studies. In research on informal help, an increasing number of publications focuses on how help and care given to older family members are related to paid employment (Bauer and Souza-Poza 2015; Choi et al. 2007; Daatland et al. 2010; Gautun and Bratt 2016; Lee and Tang 2015; Moussa 2019). In sum, the conclusion is that the correlation is negative, as caregivers are likely to work fewer hours or not be employed compared with non-caregivers. Whether or not caregivers leave the labour market altogether seems to depend on the intensity of the help and care provided, and on the relationship to the receiver. For example, Jacobs et al. (2014) find that the number of hours of personal care provision is a predictor of leaving work, whereas Carr et al. (2016) conclude that work exit is only likely to occur if the care provider and the care receiver live together. Similarly, grandparents seem to have difficulties in combining full-time paid work with regular grandchild care (Hank and Buber 2009; Lakomý and Kreidl 2015), and there is also evidence of older workers leaving paid employment when entering grandparenthood (Van Bavel and Le Winter 2013; Zanasi et al. 2020).

Volunteering among seniors has been shown to be both negatively and positively related to paid employment. Hank and Stuck (2008), Di Gessa and Grundy (2017) and Mutchler et al. (2003) all conclude that the tendency to volunteer increases after retirement, hence implying a negative relation. Others find volunteering to be positively associated with paid work in later life (Strauss 2019; van der Horst et al. 2017), but this mainly seems to hold in the case of part-time work (Carr and Kail, 2013; Mutchler et al. 2003). Carr and Kail (2013) argue that volunteering is compatible with continuing paid work on a part-time basis after leaving full-time work, both because paid activity can provide social resources facilitating engagement in other (unpaid) activities, and because those who continue in paid employment may prefer being active in general, making them more likely to volunteer as well.

How different types of *unpaid* activities are related to one another is more ambiguous. Several studies report positive links between volunteering and informal help and care (Burr et al. 2005; Di Gessa and Grundy 2017; Hank and Stuck 2008; Strauss 2019), whereas others conclude that engagement in various unpaid activities compete with (Choi et al. 2007) or are independent of each other (van der Horst et al. 2017). One possible reason why the literature deviates is that associations between different activities are commonly studied cross-sectionally, without considering the dynamic nature of the relationship (e.g. leaving one activity for another). The few studies taking a longitudinal perspective reveal that previous involvement in volunteering or informal help and care is the most important predictor of engagement in the same unpaid activity (Di Gessa and Grundy 2017; Erlinghagen 2010; Mutchler et al. 2003).

To sum up, the ‘trade-off hypothesis’ seems to receive more support than the ‘more-is more’ phenomenon, as seniors’ participation in paid work is mostly found to be negatively linked to involvement in unpaid activities. Furthermore, earlier studies confirm the importance of continuity; previous engagement in unpaid work appears to be key to participation in later life.

### The Norwegian context

The Norwegian (or Scandinavian) case is of interest from a comparative perspective because its universal and extensive welfare state is not resulting in passive citizens, but rather in high participation in both paid and unpaid work (Henriksen, et al. 2019; Jegermalm and Grassman 2009; Verbakel 2018), also in later life (Hansen et al. 2018; UNECE/European Commission 2019). Common types of unpaid activities, both voluntary work and informal help, tend to differ somewhat from the situation elsewhere in Europe. The cultural legacy of civil involvement and self-organising in Scandinavia has led to a high density of voluntary organisations within the fields of culture and leisure, whereas in continental Europe, organised volunteering in the welfare sector is more typical (Henriksen et al. 2019). In fact, according to a Norwegian study, the largest share of volunteers provides unpaid work for associations within the fields of culture and recreation (68%). Organisations related to religion, health and social services, on the other hand, attract more modest shares (9–14%) (Eimhjellen et al. 2018). The pattern is similar for older age groups (Hansen and Slagsvold 2020). The majority of volunteers in their sixties contributes sporadically or 1–2 h a week at the most, and only approximately 20% are engaged for 3 h or more every week (ibid.). This is an interesting observation, considering the recent Norwegian reform ‘A full life—all your life’, where one aim is to increase voluntary work in the health and care services by systematic recruitment of seniors. Traditionally, this sector has been left almost entirely to professional workers. Bringing volunteers in would require a firm commitment from these individuals. However, according to a recent study from Norway, few seniors are willing to commit themselves for a longer time period (i.e. 6 months) or to adjust holidays and leisure time in order to volunteer (Hansen and Slagsvold 2020).

As for informal help, the extensive availability of public care services relieves family members of intensive care responsibilities, giving them the option to concentrate on less frequent provision of practical help. This does not mean that the family wriggles out of its care responsibilities. In fact, comparative studies confirm that more men and women are involved in providing informal help and care, including grandchild care, in the Scandinavian countries than further south in Europe, but they typically invest less time (Brandt 2013; Herlofson and Hagestad 2012; Verbakel 2018). A similar cross-national pattern is found for volunteering (Hansen et al. 2018). Both characteristics, the organisation of unpaid work and time use, seem to enable Scandinavians to follow their personal interests to a greater extent, and to choose activities more out of motivation and less out of necessity to provide essential care to people with large needs (Amilon and Larsen 2020). Consequently, the competitive relationship between paid and unpaid work might be less salient in Norway than in countries outside Scandinavia.

## Data and methods

### Sample

To study associations between labour market participation, volunteering and informal help among seniors in Norway, we employ data from the Norwegian Life Course, Ageing and Generation Study (NorLAG). Three survey waves have been carried out so far (2002, 2007, 2017), including computer-assisted telephone interviews (CATI) and self-administered questionnaires that are linked to register data. We use information from Wave 2, which due to a large refresher sample is considered nationally representative (*N* = 9238 aged 40–85, response rate 61%), and Wave 3 to which 68% of earlier NorLAG participants responded (*N* = 6099, aged 50–95). In both waves, three out of four CATI respondents returned the self-administered questionnaire (Veenstra et al. 2021).

For the purpose of our analyses, we identify a subsample consisting of respondents who participated in both Waves 2 and 3 and were aged 62–75 in 2017 (*n* = 2420). The age span covers the years when workers in Norway may start to withdraw retirement pension (before turning 67, provided they have enough earnings), while they also may accumulate pension entitlements if they continue to take paid work. This age group was chosen because one of our aims is to investigate whether participation in paid work is likely to be replaced by engagement in unpaid work, and thus our respondents should have the opportunity to exit the labour market in the period between the two waves. As some of the relevant survey questions are in the self-administered questionnaire, the sample is restricted to respondents who completed this part of the survey in both waves (*n* = 1666). We exclude respondents with missing values on any variable included in the analyses, leaving us with an analytical sample of 1625 individuals.

In most surveys, particularly longitudinal studies that follow the same people over time, a challenging issue is non-response and selection bias. In NorLAG, two-thirds of the respondents aged 52–65 in the nationally representative sample of Wave 2 participated in Wave 3 (when aged 62–75). Our analytical sample, with its additional requirement that respondents completed the self-administered questionnaire in both waves, includes approximately 45% of all 52- to 65-year-olds from Wave 2. If we compare the whole Wave 2 sample (52–65) with our analytical sample, the latter has on average a higher educational level (8 pp difference), better self-reported health (6 pp difference) and were more often in paid work in Wave 2 (6 pp difference). For the tendency to provide informal help or formal volunteering, the differences are smaller and close to negligible. All in all, the selection bias is relatively modest and should not have any significant impact on the results of our analyses (see, for example, Hellevik 2016).

### Dependent and independent variables

To examine associations between paid and unpaid work over time, we have two dependent variables: informal help provision (Model 1) and formal volunteering (Model 2) (both measured in 2017). Our main independent variable measures paid work status (stability and change between 2007 and 2017). Since we also aim to study the importance of continuity, Model 1 includes informal help in 2007 and Model 2 includes volunteering in 2007. Finally, we are interested in how the two forms of unpaid work are connected. Consequently, voluntary work (2017) is included in Model 1 and help provision (2017) in Model 2.

Informal help provision is measured through several questions on help and care, including grandchild care: ‘Approximately how often do you do the following … Look after grandchildren?’, personal care: ‘Have you within the past year regularly helped someone with personal care, like eating, getting out of bed, getting dressed, or going to the toilet (not including children)?’, and practical help: ‘Have you within the past year regularly given practical help to people who you don’t live with?’. The questions were repeated in both waves. For provision of personal care and practical help, respondents had to specify in follow-up questions how often they had helped. We assign the value 1 to respondents who answered at least weekly for at least one of the three types of care provision. Formal volunteering is measured, for both waves, through a single question: ‘Approximately how often do you do the following … Voluntary work for clubs/organisations?’. We assign the value 1 to respondents who specified at least weekly.

Work status is measured through the following questions (2007 and 2017): ‘Did you do paid work for at least 1 h last week?’ and ‘If no, are you in paid work that you were temporarily absent from last week?’. A ‘yes’ answer to either question is considered as being in paid work. We separate part-time and full-time workers in 2017 by a question on whether the respondents considered their work to be part-time or full-time. Based on this information, we construct the following dummy variables: ‘Stopped working between 2007 and 2017’, ‘Not working in either 2007 or 2017’, ‘Working part-time in 2017’ and ‘Working full-time in 2017’. Most part-time and full-time workers in 2017 were also working in 2007. The few respondents (*n* = 24) who entered paid work between the two waves are included in the two latter variables depending on their work hours (part-time or full-time) in 2017.

Previous research has illustrated how the dynamic between paid and unpaid work varies with the different backgrounds and resources that people have (Wilson and Musick 1997). Hence, we control for education, subjective health status (in 2007) and living with a partner (in 2007), in addition to gender and age, which are variables that have been shown to be important for participation in both paid and unpaid work (Jongenelis et al. 2020; Komp et al. 2010). Education is measured by register data, with ‘1’ equalling college/university level. Partner status is based on a combination of register and survey information in 2007. Finally, subjective health is the respondent’s evaluation of his/her own general health (excellent/very good/good/fair/poor) in 2007, which is included as a binary variable, where ‘1’ equals the two highest values.

### Methods

We perform multivariate regression analyses using linear probability models (LPMs). An important advantage of LPM models is that their coefficients are easier to interpret than those in logistic regression. Furthermore, the statistical objections to using LPM have been shown to have little practical significance (Hellevik 2009). Additional sensitivity tests using logistic regression yield results similar to the LPMs presented here (available upon request).

## Results

### Descriptive statistics

Table 1 provides descriptive statistics for our sample of respondents aged 62–75 in 2017. Not surprisingly considering their age, close to half (49%) were in paid work in 2007 but had left by 2017. The rest are equally distributed across the three remaining categories denoting stability in work status: not being in paid work in either survey wave, working part-time in 2017, and working full-time in 2017. Turning to the two types of unpaid work (performed weekly or more often), in 2007, 33% of the sample provided informal help whereas 16% did volunteer work. For both activities, there is a small increase between the two waves but the changes are not statistically significant.

**Table 1:** Descriptive statistics (%)

Main variables	
Paid work status	
Stopped working between 2007 and 2017	49
Not working in either 2007 or 2017	17
Working part-time in 2017	16
Working full-time in 2017	18
Informal help provision (at least weekly) in 2007	33
Informal help provision (at least weekly) in 2017	37
Formal volunteering (at least weekly) in 2007	16
Formal volunteering (at least weekly) in 2017	17
Control variables	
Gender (% male)	49
Age in 2017	
62–64	23
65–66	17
67–68	15
69–70	15
71–72	15
73–75	15
Education (% college/university)	41
Living with partner 2007	79
Subjective health status (% very good/excellent) 2007	49
*N*	1625

The statistics for the control variables show that men and women are almost equally represented in the sample (49% are men). The age distribution is marginally skewed towards the youngest age group (62–64). Furthermore, 41% of the respondents have a high educational level. Finally, in 2007, 79% lived with a partner and 49% considered their health to be very good or excellent.

**Fig. 1:**
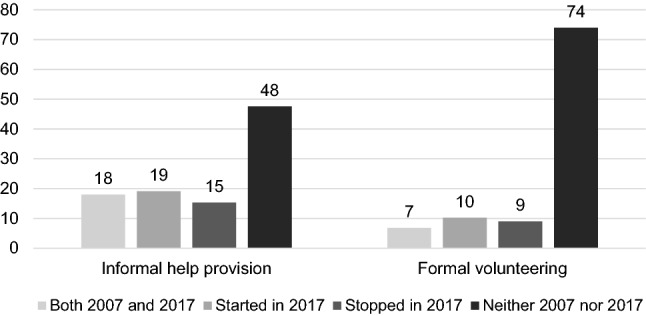
Change and stability in informal help and formal volunteering between 2007 and 2017 (%)

Table 1 reveals no substantial change in either informal help or formal volunteering between the two waves on the aggregate level. However, this apparent stability conceals the fact that people are both starting and quitting the two activities, as illustrated in Fig. 1. In the multivariate analyses, we will investigate how these individual changes in unpaid work interact with changes in paid work.

### Multivariate analyses

Table 2 presents linear regression models for the probability of providing informal help and volunteer work (at least weekly) in 2017 dependent on previous and current paid and unpaid work participation. Model 1 shows that full-time workers at follow-up (2017) have a 9% lower probability of providing informal help than those who stopped working between the two waves (reference group). Part-time workers, on the other hand, do not deviate significantly from the reference group, but additional analyses show that they are more likely to provide help than those in full-time paid work. Individuals who were not in paid work in either of the two waves do not deviate significantly from any of the other groups regarding the likelihood of providing informal help (results from the additional analyses are available upon request).

**Table 2:** Associations between paid and unpaid work: Informal help (Model 1) and formal volunteering (Model 2)

	Model 1: Informal help (at least weekly) 2017		Model 2: Formal volunteering (at least weekly) 2017	
	Coef	SE	Coef	SE
Paid work status: (ref: Stopped working between 2007 and 2017)				
Not working in either 2007 or 2017	− 0.067	0.035	− 0.038	0.027
Working part-time in 2017	− 0.014	0.034	− 0.012	0.026
Working full-time in 2017	− 0.094**	0.036	− 0.105**	0.027
Informal help provision (at least weekly) in 2007	0.246**	0.025		
Formal volunteering (at least weekly) in 2007			0.313**	0.024
Informal help provision (at least weekly) in 2017			− 0.034	0.019
Formal volunteering (at least weekly) in 2017	− 0.059	0.031		
Control variables				
Gender (ref. male)	− 0.039	0.024	0.035	0.018
Age (ref: 62–64)				
65–66	0.06	0.037	− 0.014	0.029
67–68	0.042	0.04	− 0.007	0.03
69–70	0.001	0.04	0.016	0.031
71–72	− 0.046	0.041	0.021	0.031
73–75	− 0.075	0.043	0.023	0.033
Education (ref. < college/university)	0.02	0.024	0.054**	0.019
Lives with partner	0.112**	0.029	0.003	0.022
Subjective health status (ref. < very good)	0.028	0.024	0.060**	0.018
Constant	0.240**	0.041	0.084**	0.032
Number of observations	1625		1625	
*R*^2^	0.092		0.118	

Linear probability model

**p < 0.01, *p < 0.05

The second model (Table 2) illustrates how paid work is associated with volunteering. Full-time workers (in 2017) have around 10 percentage points lower probability of doing volunteer work at least weekly compared with those who left paid work between the two waves. Neither part-time workers in 2017 nor those out of paid employment at both time points differ from individuals who left paid work between waves. In the same manner as for informal help, additional analyses indicate that part-time workers are more likely to volunteer than full-time workers. Finally, non-workers (in both 2007 and 2017) do not differ significantly from the other groups.

Engagement in the same unpaid activity 10 years earlier is of utmost importance; informal helpers in 2007 have a 25% higher probability of also helping in 2017 than those who did not provide help, whereas volunteers in 2007 have a 31% higher probability of volunteering at follow-up than those who were not engaged in such work 10 years earlier. The association between the two unpaid activities is represented by a negative coefficient in both models indicating a competing relationship, but the correlation is not statistically significant.

Turning to our control variables, there is no significant difference between men and women (in our sample of 62- to 75-year-olds) in the likelihood of regular engagement in unpaid work. Nor does age matter. Higher education and good self-reported health (in 2007) increase the propensity to be engaged in volunteering (but not in informal help). Living with a partner (2007), on the other hand, means a higher probability of providing informal help (but not volunteering) 10 years later. Both health and partner status were measured in 2007, but sensitivity tests including measures of the same variables for 2017 did not notably alter the results. Since *change* in self-reported health is also likely to be related to change in both paid and unpaid work status, we controlled for health change in additional analyses. Since the variable neither showed any significant effects nor improved the model fit, it was excluded from the final analyses.

### Interactions with previous activity in unpaid work

**Fig. 2:**
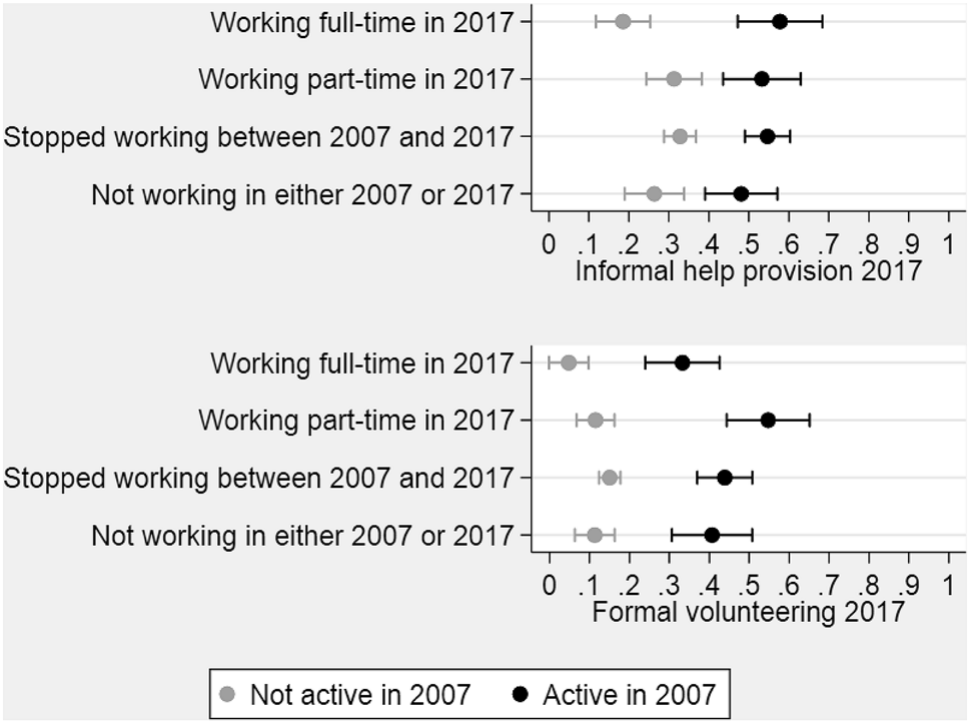
Predictions of informal help provision and formal volunteering at least weekly at follow-up, by paid work status and engagement in the same unpaid activity in 2007 (95% confidence intervals)

To investigate whether the association between paid and unpaid work varies with previous unpaid work, we performed additional analyses including interaction terms between paid work status and unpaid activity 10 years earlier. The results indicate that previous engagement is indeed relevant, but in slightly different ways for the two forms of unpaid work. Figure 2 (and Table 3 in Appendix) shows the predicted probability of informal helping and volunteering in 2017 for the work status groups when considering previous participation in the same unpaid activity: grey dots for respondents who were not active in unpaid work in 2007 and black dots for those who were active. For the previously non-active, both work exit and part-time work are associated with a higher probability of providing informal help at follow-up than full-time work. For those who were active 10 years earlier, the negative coefficient of full-time work (Table 3, Model 1 in Appendix) is balanced out by a positive interaction term, meaning that the negative association between full-time work and informal help reported in Table 2 does not apply if full-time workers provided informal help 10 years earlier.

For volunteering, the association with work status also differs according to previous voluntary engagement, but here the difference concerns part-time and not full-time work. The negative correlation between volunteering (in 2017) and full-time work compared with work exit between the waves is significant *regardless* of previous participation in volunteer work. This finding is illustrated by a non-significant interaction term between volunteering in 2007 and full-time work at follow-up (Table 3 in Appendix). For part-time work, on the other hand, earlier engagement matters. Part-time workers with a volunteer history (2007) have the highest probability of volunteering at follow-up, whereas part-time workers who did not volunteer 10 years earlier do not differ from the other groups.

## Discussion

Active ageing has become a key policy response to increasing population ageing. One measure is to encourage extended working lives. A timely question then is whether a later work exit will come at the expense of contributions from older people to voluntary work and informal help–activities that are also considered central to society.

By following a sample of Norwegian seniors (aged 62–75 in 2017) over a 10-year period (from 2007 to 2017) and investigating their engagement in paid work, informal help and formal volunteering, our study has provided insights into patterns of competition and complementarity, as well as of continuity. Overall, we find that full-time employment is associated with a lower likelihood of regular engagement in unpaid work activities than both part-time work and work exit. This is perhaps somewhat surprising, given the less demanding nature of formal volunteering and informal help in Norway (similar to the rest of Scandinavia). However, for provision of informal help, the negative association only applies to individuals who were not providing help 10 years earlier. Full-time workers involved in informal help in 2007 do not differ from part-time workers or from those who left paid work during the 10-year period. Previous unpaid engagement is not only important for the association between paid and unpaid work; it is also a crucial predictor for participation in unpaid activities in general because it increases seniors’ probability of providing both informal help and voluntary work considerably.

The negative association between full-time employment and unpaid work could be interpreted as a trade-off mechanism. However, the fact that individuals who have left the labour market are more likely to engage in unpaid work activities is not necessarily due to competition. It may simply be that when people leave paid employment, many start searching for new activities to engage in, perhaps because of the imperative of ‘active ageing’ or because they strive for some continuity in life, in line with Atchley’s theory (Atchley 1989). Another argument against an unequivocal trade-off conclusion is that the negative association between full-time work and informal help does not apply for those with previous help provision experience. Combining full-time work with informal help provision seems far less complicated if one was already engaged in informal help 10 years earlier.

Our findings indicate that part-time employment is easier to combine with regular help provision and volunteering than full-time work. The fact that work hours make a difference could be interpreted as evidence of trade-off. On the other hand, it might be more of a complementarity phenomenon since part-time workers are more likely to continue voluntary work involvement than those who have left the labour market. The interpretation of the results is also a question of causal direction, which brings us to the strengths and limitations of our study.

### Strengths and limitations

An advantage of longitudinal data is the possibility to follow the same individuals over time, and in our case, to uncover how previous paid and unpaid work behaviour and change in employment status matter for later activity patterns. Nonetheless, the causal relationship between paid and unpaid work is difficult to identify even with longitudinal data, and with our analyses we can only report statistical associations, not causal connections. Consequently, we do not know whether paid workers reduce their hours to have more time available for unpaid activities or whether long-term volunteers and informal helpers are inclined to work part-time. The same applies to the effect of work exit; engagement in unpaid work could be a result of having more time available after exit from paid work or it could be that individuals leave paid work to free up time for unpaid activities. Nor can we know whether those who continue paid work would have acted differently had they retired earlier. Different mechanisms might also be at play between various activities. While some may compete, and perhaps contribute to, a withdrawal from paid work, other activities might first become an option after retirement.

Our study of how involvement in unpaid work varies with work exit benefits from the possibility to separate seniors retiring between the two waves from those not working in either wave. This is important because people who are out of work for a longer period might have fewer resources to be active in unpaid work than more recent retirees. Yet it is not possible to distinguish individuals who have been outside the labour market for most of their life from those who left work shortly before the first interview. Both groups are included in the variable ‘Not working in either 2007 or 2017’.

An additional limitation may be that people who participate in follow-up waves of longitudinal surveys are also more likely to contribute to other socially valued activities (Abraham et al. 2009; Hermansen 2018). As reported earlier, we found only minor differences in the shares providing unpaid work in Wave 2 between Wave 3 participants and those who dropped out. Even so, the possibility of a difference between the two groups at follow-up cannot be completely disregarded. However, although sample selection might influence the share of volunteers reported from surveys, it has proven to have little relevance for the effects of variables *explaining* volunteering (Amilon and Larsen 2020; Hank and Erlinghagen 2010).

Finally, this study was limited by the survey questions and instruments, which restricted the possibility for detailed analyses of the different types of paid and unpaid work activities the respondents were engaged in, and the motivations behind their involvement. In a recent article, Van Solinge et al. (2021) identify different routes to paid and unpaid work after retirement. They find that both the need for adjustment to role loss and the search for personal development motivate post-retirement engagement. Although the Norwegian context may allow for following internal motivations more than external needs, people’s life situations, opportunities and constraints will differ, and consequently, the degree of agency will vary from person to person. To expand our understanding of seniors’ involvement in paid and unpaid activities, future research may benefit from qualitative studies, as they may provide more in-depth insights into motivations and priorities, as well as obstacles, for active ageing.

### Policy implications

In line with earlier research (e.g. Di Gessa and Grundy 2017; Erlinghagen 2010), our study confirms that the most significant predictor of regular participation in unpaid work is previous engagement in the same unpaid activity. This finding suggests that when new generations of seniors approach retirement, they do not automatically start to engage in unpaid activities in which they did not previously participate. Hence, if the goal is to increase older peoples’ contributions in voluntary work, as well as in informal help and caregiving, stimulating combinations of paid and unpaid activities in late careers may be beneficial. If seniors are already involved in unpaid work before leaving the labour market, chances are they will continue to do so.

Knowledge about available options for engaging in unpaid work proves to be important, as not being asked to participate and not receiving information about the possibilities seem to constitute important barriers to volunteering among older age groups (Hansen and Slagsvold 2020). Governments and organisations should therefore target information to men and women approaching retirement age to facilitate voluntary work among seniors. In addition, partnerships between employers and the voluntary sector could be initiated to open the door to volunteering for older workers ahead of work exit. Stimulating volunteering among younger age groups may also prove fruitful. Hogg (2016) argues that if people are socialised into a volunteer identity earlier in the life course, they may more easily resume voluntary work later in life (referred to as serial volunteers).

In Norway, similar to the other Scandinavian countries, voluntary work is commonly connected to the fields of culture and leisure, and not to the welfare sector (Henriksen et al., 2019). However, as described earlier, one of the suggestions in the recent Norwegian reform ‘A full life—all your life’ is to increase voluntary work in the health and care services by recruiting men and women entering retirement. Whether this strategy will succeed is by no means guaranteed. As our results indicate, continuity matters. Continuity is not necessarily only about carrying on with voluntary work but may also be about continuity in the same type of unpaid work, and the health and care sector is perhaps not the most appealing for individuals with no experience from this field or from voluntary work in general. Furthermore, it is a sector that requires a certain regularity and stability, which may not be what new retirees are looking for. According to a recent Norwegian study, more than half of individuals in their sixties are only willing to volunteer if they can decide for themselves when and how much time to contribute (Hansen and Slagsvold 2020). And as reported earlier, although volunteering rates are relatively high in Norway, as in the rest of Scandinavia, the time use is more restricted.

The traditionally less demanding nature of unpaid work in Norway may imply a lower threshold for engaging in volunteer work and caregiving, and for combining unpaid work with paid employment. Yet reduced work hours at the end of the paid work career (referred to as phased or gradual retirement) seem to make simultaneous engagement in the two spheres easier. Part-time work is not only advantageous because of the positive association with unpaid work; it is also presumed to be a strategy to encourage people to work longer (e.g. Wainwright et al. 2019). However, there is a risk of part-time work becoming a stepping stone into full retirement instead of leading to an extended working life (Hess et al. 2018). The causal mechanisms of part-time work, informal help and formal volunteering in late careers, and their implications for the timing of labour market exit, are issues to be further explored in future research.

## Appendix

See Table 3.

**Table 3:** Associations between paid and unpaid work: informal help (Model 1) and formal volunteering (Model 2)

	Model 1: Informal help (at least weekly) 2017		Model 2: Formal volunteering (at least weekly) 2017	
	Coef	SE	Coef	SE
Paid work status: (ref: Stopped working between 2007 and 2017)				
Not working in either 2007 or 2017	− 0.064	0.043	− 0.037	0.029
Working part-time in 2017	− 0.015	0.041	− 0.035	0.028
Working full-time in 2017	− 0.143**	0.041	− 0.103**	0.030
Interaction				
Not working in either 2007 or 2017* help 2007	− 0.001	0.067		
Working part-time in 2017* help 2007	0.001	0.070		
Working full-time 2017* help 2007	0.174*	0.071		
Not working in 2007 or 2017* vol 2007			0.006	0.067
Working part-time in 2017* vol 2007			0.144*	0.069
Working full-time in 2017* vol 2007			− 0.003	0.064
Informal help (at least weekly) in 2007	0.219**	0.035		
Formal volunteering (at least weekly) in 2007			0.288**	0.038
Informal help (at least weekly) in 2017			− 0.034	0.019
Formal volunteering (at least weekly) in 2017	− 0.058	0.031		
*Control variables*				
Education (ref. < college/university)	0.019	0.024	0.054**	0.019
Lives with partner	0.110**	0.029	0.002	0.022
Subjective health status (ref. < very good)	0.031	0.024	0.059**	0.018
Gender (ref. male)	− 0.041	0.024	0.034	0.018
Age (ref: 62–64)				
65–66	0.059	0.037	− 0.013	0.029
67–68	0.041	0.040	− 0.006	0.030
69–70	0.000	0.040	0.015	0.031
71–72	− 0.046	0.041	0.019	0.032
73–75	− 0.076	0.043	0.022	0.033
Constant	0.251**	0.042	0.088**	0.032
Number of observations	1625		1625	
*R*^2^	0.096		0.121	

Linear probability model with interaction between previous unpaid work and paid work status

***p* < 0.01, **p* < 0.05
